# Transcriptomics and the Mediterranean Diet: A Systematic Review

**DOI:** 10.3390/nu9050472

**Published:** 2017-05-09

**Authors:** Luis V. Herrera-Marcos, José M. Lou-Bonafonte, Carmen Arnal, María A. Navarro, Jesús Osada

**Affiliations:** 1 Departamento de Bioquímicay Biología Molecular y Celular, Facultad de Veterinaria, Instituto de Investigación Sanitaria de Aragón-Universidad de Zaragoza, E-50013 Zaragoza, Spain; l.vte.herrera@gmail.com (L.V.H.-M.); angelesn@unizar.es (M.A.N.); 2 Instituto Agroalimentario de Aragón, CITA-Universidad de Zaragoza, E-50013 Zaragoza, Spain; mlou@unizar.es (J.M.L.-B.); arnal@unizar.es (C.A.); 3 Departamento de Farmacología y Fisiología, Facultad de Ciencias de la Salud y del Deporte, Instituto de Investigación Sanitaria de Aragón-Universidad de Zaragoza, E-22002 Huesca, Spain; 4 CIBER de Fisiopatología de la Obesidad y Nutrición, Instituto de Salud Carlos III, E-28029 Madrid, Spain; 5 Departamento de Patología Animal, Facultad de Veterinaria, Instituto de Investigación Sanitaria de Aragón-Universidad de Zaragoza, E-50013 Zaragoza, Spain

**Keywords:** transcriptome, microarray, gene expression profile, Mediterranean diet, olive oil

## Abstract

The Mediterranean diet has been proven to be highly effective in the prevention of cardiovascular diseases and cancer and in decreasing overall mortality. Nowadays, transcriptomics is gaining particular relevance due to the existence of non-coding RNAs capable of regulating many biological processes. The present work describes a systematic review of current evidence supporting the influence of the Mediterranean diet on transcriptomes of different tissues in various experimental models. While information on regulatory RNA is very limited, they seem to contribute to the effect. Special attention has been given to the oily matrix of virgin olive oil. In this regard, monounsaturated fatty acid-rich diets prevented the expression of inflammatory genes in different tissues, an action also observed after the administration of olive oil phenolic compounds. Among these, tyrosol, hydroxytyrosol, and secoiridoids have been found to be particularly effective in cell cycle expression. Less explored terpenes, such as oleanolic acid, are important modulators of circadian clock genes. The wide range of studied tissues and organisms indicate that response to these compounds is universal and poses an important level of complexity considering the different genes expressed in each tissue and the number of different tissues in an organism.

## 1. Introduction

The traditional Mediterranean diet (considered by United Nations Educational, Scientific and Cultural Organization (UNESCO) as a heritage of humanity) used to be the food consumption pattern in countries around the Mediterranean basin during the decade of the 1960s. Epidemiological and clinical studies have established the health benefits of the Mediterranean diet, which is associated with a lower incidence of atherosclerosis, cardiovascular diseases, some types of cancer, and overall mortality [1,2].

The Mediterranean diet is largely vegetarian in nature, and olives and olive oil derived from the olive tree (*Olea europea*) are important components. The medicinal properties of its leaves and fruit have been known since antiquity. In particular, olive oil consumption has been associated with a decreased risk of cardiovascular disease and certain cancers [3,4]. It is now well-established that, in olives and extra virgin olive oil (EVOO), not only the monounsaturated fatty acid constituents but also the minor phenolic components and other phytochemicals have important health benefits [5]. In this regard, research over the past decade has highlighted the beneficial effects of a range of phenolic compounds [6], particularly for cardiovascular disease, metabolic syndrome, and inflammatory conditions. In acute or long-term administration, both in vivo and in vitro studies have shown that EVOO components have positive effects on metabolic parameters, such as plasma lipoproteins, oxidative damage, inflammatory markers, pro-thrombotic markers, platelet function, and antimicrobial activity. These bioactive compounds may influence gene expression at different levels such as transcription, maturing and stability of RNAs, their translation into proteins, and post-translational modifications [7]. Accordingly, they may regulate pathways and proteins related to those pathological entities. However, the underlying mechanisms remain unknown.

**Figure 1:**
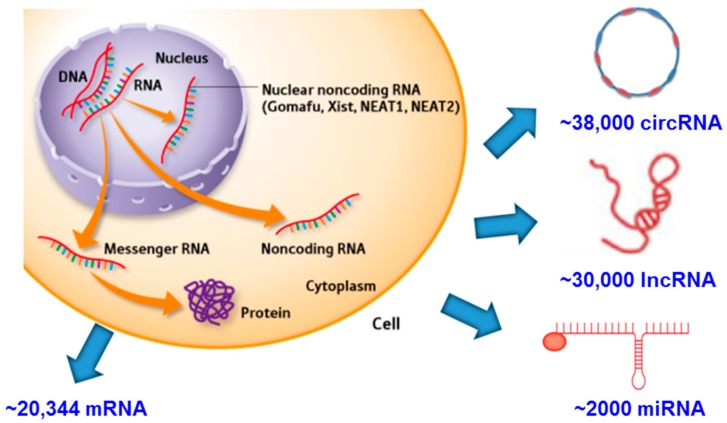
Current perspective of potential types of RNA in a hypothetical human cell. Prepared using information from [9,10,11,12].

Transcriptional studies have been particularly addressed due to RNA constant chemical properties of water-solubility and the ability to recognize complementary molecules of RNAs, in contrast to proteins where such uniform patterns do not exist. In an attempt to better understand the interactions between nutrients and gene expression, high throughput gene expression using DNA chips or microarrays has been introduced and applied to analyze transcriptomes. In contrast to genomic studies, there is not a single transcriptome for an organism but one for each cell, and it may change in certain environmental circumstances [8]. The discovery of a large number of non-coding RNAs (approximately 70,000) (Figure 1) with regulatory functions opens a new field of study for nutrient action and emphasizes the study of transcriptomics as an end-point of regulatory control. In this review, special attention will be paid to the changes in a number of tissues considering the different transcriptional programs that they have undergone [9], and that their responses to nutritional stimuli are established upon diverse transcription factors and may have distinct physiological purposes [8].

**Figure 2:**
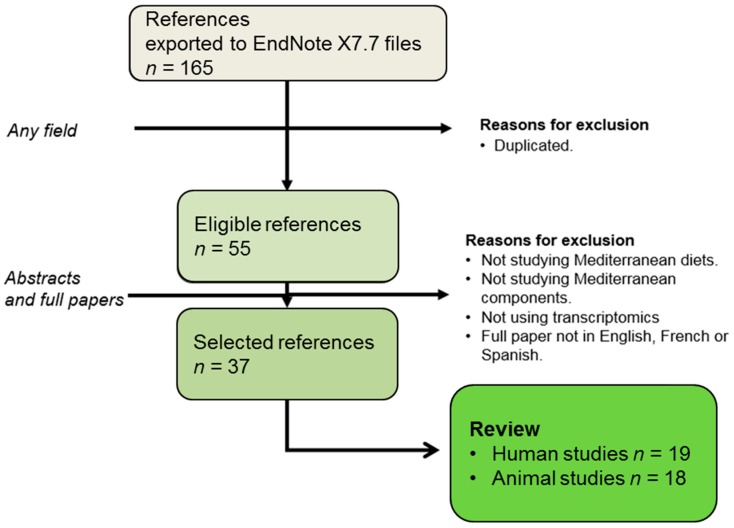
Flow chart displaying the stages used to select the references considered. EndNote X7.7 (Bld 9325 Thomson Reuters: New York, NY, USA, 2016).

The present review has adhered to systematic review guidelines [13], and the keywords used to search PubMed [14] are reflected in Table 1. It covers the up-to-date knowledge of nutrigenomic studies dealing with the Mediterranean diet, olive oil, or its components. It identified 165 hits from November 1945 to 7 April 2017. The search was refined by eliminating duplicate documents. The 53 papers obtained were critically reviewed to verify whether they analyzed transcriptomics and the Mediterranean diet or its components (Figure 2). Documents that failed to meet this criterion were excluded. Thus, this review covers the works related to the effects of Mediterranean diet and transcriptomics in 37 papers.

## 2. Technical Considerations

Table 2 reflects 37 published studies corresponding to 34 transcriptomic analyses. In most of them, the tool of choice was the microarray, and Affymetrix platforms were those most preferred. Agilent, Ilumina, Applied Biosystems, and home-made low-density arrays were less frequently used. Just four papers used RNA seq [15,16,17,18] and there was one serial analysis of gene expression [19]. Equally variable was the question employed in the search, as reflected in the intervention column. This review will be categorized according to the Mediterranean diet components. Human peripheral blood mononuclear cells (PBMC) and a wide range of tissues were employed in these studies. Another complication is the consequence of rapid updating, with increasing numbers of transcripts added in recent versions. Assuming all these limitations, the information gathered should be considered in progress, but a glimpse of a powerful tool to delineate nutritional components is clearly envisioned.

## 3. Transcriptional Analysis of Mediterranean Diet Consumption or Its Components in Human Studies

PBMC play a crucial role in the initiation and progression of atherosclerosis [52] and are easily accessible cells. For these reasons, the majority of human transcriptional studies have been performed using them as samples with different experimental designs and types of subjects. Adipose tissue biopsies and other cells of human origin have also been employed in order to test in vitro actions.

### 3.1. Influence of a Mediterranean Diet

A six-week intervention of a Mediterranean-inspired diet was able to reduce the established biomarkers of inflammation and resulted in a trend toward normalizing the microbiota in Crohn’s disease patients. A transcriptomic approach showed that the cumulative effect of small changes in many genes combined to have a beneficial effect [21]. In another experiment, the effects of a Mediterranean-type diet, and the replacement of saturated fatty acids (SFA) with monounsaturated fatty acids (MUFA) in a Western-type diet, were tested in abdominally overweight men and women allocated to an eight-week, completely controlled SFA diet (19% daily energy as SFA), a MUFA diet (20% daily energy MUFA using refined olive oil), or a Mediterranean diet (21% daily energy MUFA using EVOO). Consumption of the MUFA and Mediterranean diets, compared with the SFA diet, decreased the expression of oxidative phosphorylation genes, plasma connective tissue growth factor, and apolipoprotein B concentrations. Compared with the Mediterranean and SFA diets, the MUFA diet changed the expression of genes involved in B-cell receptor signaling and endocytosis signaling. Participants who consumed the Mediterranean diet had lower concentrations of proinflammatory proteins at the end of the intervention. Thereby, the replacement of SFA with MUFA may improve health, by reducing metabolic stress and oxidative phosphorylation activity in PBMC [22]. The Mediterranean diet may have additional antiatherogenic effects by lowering proinflammatory plasma proteins due to other biological active compounds [53].

The influence of postprandial ingestion of a single dose of olive oil (50 mL) on gene expression was analyzed in PBMC cells from six healthy male volunteers. Compared with the fasting state, upregulated expressions were observed in genes related to metabolism, cellular processes, and cancer, and downregulation was described for genes related to atherosclerosis (e.g., *USP48* and *OGT*) and associated processes such as inflammation (e.g., *AKAP13* and *IL*-*10*) and DNA damage (e.g., *DCLRE1C* and *POLK*) [26].

The long-term (3-month) effect of a traditional Mediterranean dietary pattern on the whole transcriptomic response was explored in a subsample (*n* = 34) of the *Prevención Con Dieta Mediterránea* (PREDIMED) study, where participants consuming a low-fat diet as a control group were compared to two intervention groups assigned to a traditional Mediterranean diet supplemented with either virgin olive oil or nuts. The Mediterranean diet with virgin olive oil elicited changes in 241 genes (139 upregulated and 102 downregulated genes), while the supplementation with nuts modified the response of 312 genes (165 upregulated and 147 downregulated genes), and the low-fat diet changed the expression of 145 genes (100 upregulated and 45 downregulated genes) compared to the initial status. Using functional annotation analysis, 12 pathways were differentially expressed, and 43% of pathways were modulated by both Mediterranean interventions; the most prevalent pathways were related to atherosclerosis and hypertension (i.e., *ADRB2*, *IL7R*, *IFNgamma*, *MCP1*, *TNFalpha*) and were often associated with an improvement in systemic markers for oxidation and inflammation [7,27]. Thereby, these changes in the transcriptomic response of genes related to cardiovascular risk may be crucial in the mechanisms of Mediterranean diet benefits.

### 3.2. Influence of Monounsaturated vs. Saturated Fatty Acid Containing Diets

The influence of the nature of administered fat on the adipose tissue transcriptome was studied in an 8 week parallel controlled-feeding trial using abdominally overweight subjects at risk of metabolic syndrome. The inclusion of refined olive oil to provide 20% of their intake as monounsaturated fatty acids compared to the 11% of the saturated diet led to a more anti-inflammatory gene expression profile [20]. No information on the source of saturated fat was provided. Considering that adipose tissue plays an interesting role in lipid metabolism and inflammation, this replacement of dietary saturated fat with a monounsaturated fatty acid diet could prevent adipose tissue inflammation and may reduce the risk of inflammation-related diseases where this tissue is particularly relevant e.g., metabolic syndrome.

The different postprandial response comparing SFA and MUFA was tested in a cross-over study in which 17 lean and 15 obese men received two 95 g fat shakes differing in the type of fat, and their gene-expression profiles were compared in the fasting and 4 h postprandial states. During fasting, 294 genes were differently expressed between lean and obese people. The fat challenge increased differences to 607 genes after SFA (palm oil) and 2516 genes after MUFA coming from high-oleic acid sunflower oil. In both groups, SFA decreased expression of cholesterol biosynthesis and uptake genes and increased cholesterol efflux genes and MUFA increased inflammatory genes such as Toll-like receptor signaling and interleukin 8-chemokine receptors 1/2 IL8-CXCR1/2 pathways and peroxisome proliferator-activated receptor alpha PPAR-alpha targets involved in beta-oxidation [25]. These results indicate that a fat challenge magnifies differences in health status of subjects, especially after MUFA, and these types of fatty acids exert distinct effects on lipid handling pathways in PBMC cells.

The effect of the type of fat on gene expression in human coronary artery smooth muscle cells was studied by incubating them with postprandial triglyceride-rich lipoproteins prepared from the plasma of healthy volunteers who had received meals enriched in refined olive oil, butter, or a mixture of vegetable and fish oils. Sixty-six genes were regulated by the incubation of lipoproteins obtained from the butter group, 55 after olive oil consumption, and 47 with vegetable and fish oils. Lipoproteins obtained from subjects consuming butter and vegetable and fish oils induced the expression of genes involved in inflammation, particularly macrophage-inhibiting cytokine-1, while those coming from the olive oil group promoted a less atherogenic gene profile [34].

The effect of the source of dietary fat on prostate cancer progression was tested in male immunodeficient mice injected with LAPC-4 human prostate cancer cells. Two weeks after injection, mice were randomized to Western diets (35% kilocalories from fat) containing the different fat sources—fish oil, olive oil, corn oil, and animal fat—and euthanized when tumor volumes reached 1000 mm^3^. The fish oil group had decreased gene expression in pathways related to mitochondrial physiology and insulin synthesis/secretion, slowed tumor growth, and improved survival compared with that of the mice consuming the other diets [33]. Therefore, the type of dietary fat consumed may be important for controlling tumor progression.

Thus, the fatty acid composition of diets modified gene expression patterns of adipocytes, PBMC, coronary artery smooth muscle, and human prostate cancer cells. The inclusion of MUFA from olive oil led to a program with lower expression of inflammatory genes and, consequently, to a less atherogenic profile, and tumor progression was controlled when compared to other sources of fat.

### 3.3. Influence of Monounsaturated vs. n-3 Fatty Acid Containing Diets

The effect of six week supplementation with either olive oil, eicosapentaenoic acid (EPA), or docosahexaenoic acid (DHA) on gene expression was tested in subjects with mild elevation in plasma lipoprotein-phospholipase A2 (Lp-PLA_2_) at baseline and six weeks after receiving olive oil 6.0 g/day (*n* = 16), EPA 1.8 g/day (*n* = 16), or DHA 1.8 g/day (*n* = 18). Neither DHA nor olive oil produced changes, but EPA significantly affected gene expression in the following pathways: (1) interferon signaling; (2) receptor recognition of bacteria and viruses; (3) G protein signaling, glycolysis, and glycolytic shunting; (4) S-adenosyl-l-methionine biosynthesis; and (5) cyclic adenosine monophosphate (cAMP)-mediated signaling including cAMP responsive element protein 1 (CREB1), as well as many other individual genes, such as hypoxia inducible factor 1 alpha subunit(HIF1A) [29]. Thus, EPA supplementation was associated with significant effects on gene expression involving the interferon pathway, as well as downregulation of *CREB1* and *HIF1A*, which may relate to its beneficial effect on cardiovascular risk reduction.

### 3.4. Influence of the Administration of Olive Oil Enriched in Phenolic Compounds

These compounds have been the focus of many transcriptomic analyses. Using PBMC, the presence of virgin olive oil phenolic compounds was tested in 20 patients with metabolic syndrome consuming two virgin olive oil-based breakfasts with high and low (398 and 70 mg/kg, respectively) contents of phenolic compounds in a double-blinded, randomized, crossover design. Olive oil phenolic compounds differentially expressed 98 genes (19 upregulated and 79 downregulated). Among these, several genes that seemed to be involved in inflammatory processes mediated by transcription factor NF-kappaB, activator protein-1 transcription factor complex AP-1, cytokines, mitogen-activated protein kinases (MAPK), or arachidonic acid pathways were repressed by phenolic compounds, thereby switching the activity of PBMC to a less deleterious inflammatory profile [23]. Recently, D’Amore et al. used a similar approach in healthy subjects and in patients with metabolic syndrome. They reported that phenolic compounds modulate mRNA and miRNA levels participating in metabolism, inflammation, and cancer. They confirmed the switch of PBMC to a less deleterious inflammatory phenotype with more information, since they used an advanced version of microarray and the inclusion of miRNAs. Furthermore, they found a more pronounced effect in healthy subjects when compared to patients [24]. These results indicate that changes in gene expression are part of the postprandial protective action of olive oil consumption and phenolic compounds exert an influence. In addition, these studies pose the question that the response may change depending on the studied population.

A mid-term (3 weeks) intervention with feasible daily doses of 50 mL explored the genes that responded to virgin olive oil consumption in order to ascertain the molecular mechanisms underlying its beneficial action in the prevention of atherosclerosis. To this end, gene expression profiles from healthy individuals were examined after moderate and regular consumption of virgin olive oil, as the main fat source in a diet controlled for antioxidant content. The response to virgin olive oil consumption was confirmed for 10 upregulated genes (*ADAM17*, *ALDH1A1*, *BIRC1*, *ERCC5*, *LIAS*, *OGT*, *PPARBP*, *TNFSF10*, *USP48*, and *XRCC5*) [28].

Overall, these studies indicate that the consumption of virgin olive oil with phenolic compounds, either in a postprandial regimen or over a relatively short period, influences the expression of genes related to atherosclerosis development and progression, and that this effect is observed at the moderate doses consumed in this dietary pattern. Whether this action is due to a particular phenolic compound or to a combination of them has not been addressed. The translation into proteins has not been analyzed.

### 3.5. Influence of the Administration of Isolated Phenolic Compound Extracts

This possibility has been explored only at the cell culture level. Indeed, EVOO extracts composed of hydroxytyrosol, secoiridoids, and lignans were tested in vitro in a colon cancer cell line engineered to overexpress estrogen receptor beta (HCT8-beta8). The extracts showed an antiproliferative effect through the interaction with estrogen-dependent signals involved in tumor cell growth. Specifically, the ability of the extracts to inhibit cell proliferation was superimposable to the activation of the estrogen receptor beta, similar to what was observed after 17beta-estradiol incubation [30]. Using HER2-gene amplified JIMT-, a cell line used to explore resistance against HER1/2-targeted drugs, the anti-cancer activity of EVOO phenolic extracts from 14 Spanish single-variety oils positively correlated with the phenolic index (i.e., total content of phenolic components) and with a higher content of secoiridoids, rather than lignans. These changes using a transcriptome approach were found to be associated with changes at the level of the cell cycle and p53 pathways. Indeed, phenolic extracts differentially modulated the expression of stress-sensing, G2-M check-point-related GADD45 genes, and of p53-related *CDKN1A*, *CDKN1C*, and *PMAIP*-*1* genes. Secoiridoid-rich extracts inhibited mitosis by promoting gap2-mitosis cell cycle arrest, induced hyperacetylation of histone H3 at lysine, and activated the mitogen-activated protein kinases MEK1 and p38. Thus, secoiridoids may be adjuvants in breast cancer therapy, particularly in refractory to HER-targeting therapies and may facilitate the development of a series of new agents to combat this cancer [31]. In fibroblasts, phenolic extracts mainly containing the secoiridoids oleuropein aglycon and decarboxymethyl oleuropein aglycon were found to activate endoplasmic reticulum stress and the unfolded protein response, spermidine and polyamine metabolism, and sirtuin-1 and NRF2 signaling. These secoiridoids also activated AMP-activated protein kinase and suppressed crucial genes involved in the Warburg effect and the self-renewal capacity of cancer cells triggering anti-aging transcriptomic signatures [32]. In bone-marrow mesenchymal stem cells, oleuropein was found to upregulate the expression of 60% of adipogenesis-repressed genes [19].

Hydroxytyrosol, the main phenolic compound in minor constituents of olive oil, has been shown to be a potent antioxidant and has anti-atherogenic and anti-cancer properties. Using genome-wide mRNA-seq, the treatment of keratinocytes with 20 µM hydroxytyrosol resulted in the upregulation of numerous antioxidant proteins and enzymes, including heme oxygenase-1, glutaredoxin, and glutathione peroxidase. This may account for the reduction in oxidative stress and suggests a mechanism for the chemoprevention of cancer by hydroxytyrosol. Alteration in the expression of transcription factors such as *STAT3*, *STAT6*, *SMAD7*, and *ETS*-*1* may also contribute to the anti-cancer and anti-inflammatory effects. Furthermore, the downregulation of the telomerase reverse transcriptase subunit, observed in the erythroleukemic cell line K562 treated with this compound [15], together with changes in the complement system, the Warburg effect, and chromatin remodeling, are other candidate genes involved in the prevention of cancer by hydroxytyrosol [16].

These in vitro studies clearly indicate that extracts enriched in hydroxytyrosol, secoiridoids, and lignans are able to inhibit cell proliferation by modulating cell cycle expression. Hydroxytyrosol itself has been shown to exert transcriptional changes related to transcription factors. However, the action of secoiridoids cannot be neglected and a combined action is expected. Direct proof of their action in controlled clinical trials is still missing.

## 4. Transcriptional Analysis of Mediterranean Diet Consumption or of Its Components in Non-Human Studies

Several animal models have been tested, as well as a wide range of tissues. In these experimental settings, not only was the influence of olive oil studied but that of its minor components as well. In these experiments, it was possible to delineate the physiological role of genes with unknown function as well.

### 4.1. Influence of Monounsaturated vs. n-3 and n-6 Polyunsaturated Fatty Acid Containing Diets

Japanese flounders were used to identify hepatic genes involved in adaptation to *n*-3 unsaturated fatty acid-depleted diets and the possibility of breeding them to be resistant to this condition. They were fed diets supplemented with fish oil, linseed oil (LO), or olive oil for 6 weeks. The latter two groups showed significantly retarded growth, lower feed intake, lower protein efficiency ratio, and lower hepatosomatic index. As a result of the administration of a diet with *n*-3 highly unsaturated fatty acid deficiency, 169 transcripts changed their expression, particularly those involved in signal transduction (23.2%), cellular processes (21.1%), metabolism (including glucose, lipid, and nucleotide; 15.5%), transport (11.3%), regulation of transcription (10.5%), and immune response (4.2%). Several genes encoding serine/threonine kinases (such as protein kinase C and calmodulin-dependent kinase), nuclear hormone receptors (such as vitamin D receptor, retinoic acid receptor) and receptors for cytokines (bone morphogenic protein and transforming growth factor beta) were also influenced. Among 169 transcripts, 57 genes were upregulated and 38 genes were downregulated in the LO group, whereas in the olive oil group, nine genes were upregulated and 87 genes were downregulated [36]. These results indicate that changes in gene expression induced by *n*-3 deficiency are not restored by the supplementation of LO or olive oils.

The influence of dietary fat on colon tumor formation was explored in Sprague-Dawley rats assigned to three dietary groups differing only in the type of fat (corn oil/*n*-6 PUFA, fish oil/*n*-3 PUFA, or olive oil/*n*-9 monounsaturated fatty acid) receiving the carcinogen azoxymethane for 12 h and 10 weeks. Only the consumption of *n*-3 PUFA exerted a protective effect at the initiation and promotional stages, estimated as DNA adduct formation and aberrant crypt foci, respectively. Colonocyte gene expression profiles were different at both the initiation and promotional stages of tumor development since the number of differentially expressed genes in each of the three diets increased with the progression of colon cancer, but each dietary lipid source exhibited its own unique transcriptional profile. The authors observed that the chemopreventive effect of fish oil was due to the direct action of *n*-3 PUFA and not to a reduction in the content of *n*-6 PUFA [43], andthey [44] found that the mediators of stem cell homeostasis, e.g., ephrin B1 and bone morphogenic protein 4, were involved. In other carcinogenesis models, the effects of a low-fat, high-corn-oil, or high-EVOO diet from weaning or from induction on the susceptibility of the mammary gland to experimental malignant transformation were tested. Whereas rats consuming EVOO modified the expression of metabolism genes, those consuming corn oil showed downregulated expression of genes related to the immune system and apoptosis, and increased proliferation and lower apoptosis in the mammary glands, thereby favoring mammary tumorigenesis [50]. Recently, Govindarajah et al. [18] tested the effects of prenatal high-fat diet (olive oil, butterfat, or safflower) exposure on cancer susceptibility in prepubertal mammary glands of rats postnatally treated with dimethylbenz[alpha]anthracene (DMBA). High-fat diet exposure significantly increased the tumor volume. Transcriptome profiling identified 43 differentially expressed genes in the mammary glands of the butterfat group as compared with controls. Differences in the expression patterns of *Lrrn1*, *NF1*, *DBF4*, *Cadm4*, *Tmem45b*, and *Btn1a1*, among high-fat diet groups, supported the existence of certain diet-dependent cancer modifier networks underlying differential susceptibility to mammary cancer risk in adult life. Collectively, these findings suggest that colon and mammary gland tumorigenesis is modulated by dietary fat and involves changes in gene expression.

A short-term—two-week—nutritional effect of dietary fat on the liver mRNA expression profile was tested in rats consuming isocaloric diets containing 22% of calories as butter or olive and fish oils together with butter (10%, 6%, and 6% of the total energy, respectively). The latter diet induced 72 and inhibited 180 genes related to lipolysis or lipogenesis and newly identified responders from other metabolisms. Some of these genes were also reported to be similarly modulated by the action of fibrates, but without the complete gene pattern of these agents [46]. Likewise, the increased expression of many PPAR-dependent enzymes mediating fatty acid oxidation followed the substitution of dietary monounsaturated or polyunsaturated fatty acids (olive or menhaden oils as 40% of calories) for carbohydrates in corpulent leptin receptor-deficient rats. This fact, together with the reduced hepatic expression of sterol regulatory element-binding protein-1c (SREBP-1c) and the enzymes related to lipid synthesis, could explain the observed decrease in hepatic triglyceride content and lipogenesis [45]. A three-week regimen was selected to explore the influence of the amount of fat (5% and 70%), provided by olive, corn, and echium oils on gene expression in male Sprague-Dawley rats. At the low fat content, compared with the corn oil group, differences in hepatic gene expression signatures associated with greater fatty acid synthesis, carbohydrate-responsive element-binding proteins, and SREBP-1c signaling, and increased fatty acid transport was observed in the olive oil group. With the higher fat diet (70%), when compared to the corn oil group, rats receiving the olive oil-enriched diet showed increased antioxidant pathways and lower expression of genes linked to inflammation and fibrosis, despite the increased macrosteatosis, but with no further hepatic damage [48]. Thereby, a Mediterranean diet high in olive oil may reduce the risk of non-alcoholic fatty liver disease progression to nonalcoholic steatohepatitis.

The effects of 3% of dietary fat from different sources, i.e., beef tallow, soybean oil, olive oil, and coconut oil, on gene expression in Longissimus dorsi muscle was explored in growing-finishing crossbred pigs (LandracexLarge WhitexDuroc). The type of dietary fat modified the fatty acid composition, and six genes involved in the insulin signaling pathway were differentially expressed depending on the dietary group. In particular, the genes encoding the cAMP-dependent protein kinase regulatory, type II, alpha, and the catalytic subunit of protein phosphatase 1, beta isoform showed the highest expression levels in the olive oil group [51]. Thus, a slight change in the source of fat is able to induce changes in gene expression in pig muscle.

Even more, not all MUFA behave alike. Indeed, using a transcriptomics approach, Yang et al. found that long-chain MUFA are particularly active at upregulating PPAR signaling pathways in the liver of low-density lipoprotein receptor-deficient mice [17].

### 4.2. Influence of the Administration of Olive Oil Enriched in Phenolic Compounds

The challenge for the liver of the postprandial phase was characterized by analyzing the gene expression profile in response to a bolus of 5 mL of EVOO in male Wistar rats. Compared with the fasting state, hepatic *A2m*, *Slc13a5*, and *Nrep* mRNA expressions were significantly modified at four and eight hours after fat ingestion and showed the highest significant associations with postprandial plasma triglycerides [47]. These results highlight the rapid changes in hepatic gene expression produced by a postprandial regimen and the discovery of new proteins involved in the process.

Middle-aged C57Bl/6J mice fed for 6 months with EVOO rich in phenols (6 mg/kg/day) showed cognitive and motor improvement compared to controls fed the same olive oil deprived of phenolic compounds. To evaluate whether these behavioral modifications were associated with changes in gene and miRNA expression, two areas involved in cognitive and motor processes were chosen: the brain cortex and cerebellum, and their gene and miRNA profiling were analyzed by microarrays. Most of the gene expression changes were restricted to the cerebral cortex. Compared to low phenolic content administration, the genes modulated by aging were mainly downregulated by high phenolic olive oil, and this diet significantly upregulates genes associated with synaptic plasticity and with motor and cognitive behavior, such as *Notch1*, *Bmps*, *Ngfr*, *Glp1r*, and *Crtc3*. The agrin pathway was also significantly modulated and miRNAs were mostly upregulated in old animals consuming low-phenolic olive oil compared to young. However, mice fed the phenolic-enriched olive oil displayed a miRNA expression profile similar to that observed in the cortex of young mice and sixty-three miRNAs were significantly downregulated, particularly *mir*-*484*, *mir*-*27*, *mir*-*137*, *mir*-*30*, *mir*-*34*, and *mir*-*124* [38]. These results indicate that a food rich in phenols can modulate, at the molecular level, the expression of genes and miRNAs involved in neuronal function and synaptic plasticity, along with cognitive, motor, and emotional behavior.

### 4.3. Influence of the Administration of Isolated Phenolic Compound Extracts

Since tyrosol was found to increase the lifespan of *Caenorhabditis elegans*, a transcriptomic approach was designed to search for candidate genes or signaling pathways potentially involved in its effects on longevity. Analyses of microarrays identified 208 differentially expressed genes (206 up- and two downregulated) when comparing nematodes treated with or without the compound. The regulation of growth, transcription, reproduction, lipid metabolism, and body morphogenesis were target genes [35]. The pattern of expression found overlapped with that elicited by quercetin and tannic acid, known lifespan enhancers in this model, and raise the notion of key cellular mechanisms directly related to longevity and the possibility of manipulating them by using these compounds.

As already mentioned, adipose tissue, where inflammation, oxidative stress, and the secretion of adipocytokines contribute to cardiovascular risk, is a target organ in cardiometabolic prevention. Long-term supplementation to mice of hydroxytyrosol at nutritionally-relevant doses, i.e., 0.03%, modulated the antioxidant network in the adipose tissue, as mediated by glutathione and associated enzymes, and reduced circulating leptin [37]. These effects, also confirmed in cultured adipocytes, indicate that low, physiological concentrations of hydroxytyrosol were able to blunt the alteration of oxidative stress.

In a rat model of experimental mammary carcinoma induced by DMBA, the administration of 0.5 mg/kg hydroxytyrosol for six weeks was found to inhibit the experimental mammary tumor growth and proliferation rate, with results comparable to those of doxorubicin. It also altered the expression of genes related to apoptosis, cell cycle, proliferation, differentiation, survival, and transformation pathways. It modified the Wnt signaling pathway [49].

Altogether, these studies provide evidence that tyrosol and hydroxytyrosol are able to control growth, oxidative stress, and differentiation in different experimental models.

### 4.4. Influence of the Administration of Other Minor Components of Virgin Olive Oil

In apolipoprotein E (*Apoe*)-deficient mice, an olive oil-enriched diet reduced fatty liver disease, and the degree of hepatic steatosis was associated with hepatic *Fsp27* and *Syt1* expressions [54]. The influence of the unsaponifiable fraction in virgin olive oil on hepatic gene expression was also tested in these mice using two isoenergetic, isonitrogenous diets containing either 10% (*w/w*) refined olive oil or olive oil enriched with the unsaponifiable fraction. Eleven genes with remarkably modified expression (signal log_2_ ratio >3 or <−3) were selected and confirmed by quantitative polymerase chain reaction. Orosomucoid and serum amyloid A_2_ were upregulated by the unsaponifiable fraction to a variable extent, depending on the mouse genetic background, in the absence of hepatic steatosis and inflammation. *Fabp5* and *Mt2* were also strongly upregulated. Several proteases were markedly suppressed by the unsaponifiable-fraction-enriched olive oil diet. The findings indicate that these genes are good targets of the unsaponifiable fraction of olive oil [40]. Two studies were carried out to investigate the effects of two triterpene components of the unsaponifiable fraction, maslinic and oleanolic acid, both present in virgin olive oil at concentrations ranging from 3 to 49 and 3 to 78 mg/kg, respectively. The effect of maslinic acid on hepatic gene expression was tested in *Apoe*-deficient mice with a C57BL/6J genetic background receiving 10% (*w/w*) fat diets. In mice receiving the maslinic-enriched diet, *Cyp2b9*, *Cyp2b13*, and *Dbp* expressions appeared to be significantly increased, and *Marco*, a gene strongly upregulated by the absence of APOE, was significantly decreased. *Dbp* was upregulated to an extent depending on the genetic background of the mice and was negatively associated with the expression of *Marco*. Thus, maslinic acid is active in controlling hepatic gene expression and some of its effects are modulated by the presence ofAPOE [42]. The other triterpene, oleanolic acid, administered to *Apoe*-deficient mice, increased the hepatic area occupied by lipid droplets with no change in oxidative stress. *Bmal1* and the other core component of the circadian clock, *Clock*, together with *Elovl3*, *Tubb2a*, and *Cldn1* hepatic expressions, were significantly increased, while *Amy2a5*, *Usp2*, *Per3*, and *Thrsp* were significantly decreased. *Bmal1* and *Cldn1* expressions were positively associated with lipid droplets. Increased *Clock* and *Bmal1* expressions were also observed in F344 rats, but not in *Apoa1*-deficient mice. These results indicate that the core liver clock components, *Clock*-*Bmal1*, are targets of oleanolic acid independently of the diets provided, and this compound requires APOA1-HDL for its hepaticaction [41]. Overall, these results highlight the important biological effects of the terpenes that are constituents of the unsaponifiable fraction of virgin olive oil, whereas oleanolic and maslinic acids are particularly active in controlling the circadian clock and inflammation genes, respectively.

## 5. Outlook

Future studies are in progress. For instance, “New dietary strategies addressing the specific needs of elderly population for a healthy aging in Europe” (NU-AGE), an interdisciplinary European research network including research centers on nutrition and aging and food industries, will explore the transcriptome of elderly people following the Mediterranean diet for one year [55]. This and other designs will complete our understanding of the complex interaction of nutrients and gene expression.

## 6. Conclusions

**Figure 3:**
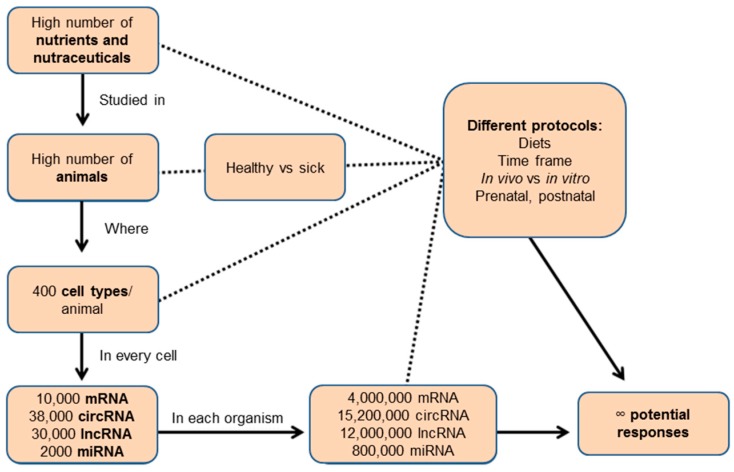
Potential transcriptomic changes in different cells upon exposure to the Mediterranean diet.

Nutrition is undoubtedly the most important environmental factor that living beings face. On this basis, a complex process of adaptation from a genomic point of view is expected. Therefore, nutritional regulation of many genes will exist, and its failure may be behind the development of many diseases. The large number of gene expression changes observed in nutritional interventions due to the increased sensitivity of using transcriptomics, as compared to well-established biomarkers, opens up a new era of dietary intervention studies. A representative overview of the magnitude of this task is represented in Figure 3. To accomplish this endeavor, an international consortium is required that should be committed to four goals: a well-defined composition of chemical compounds in food; bioavailability studies to characterize in vivo levels of minor components; characterization of all types of RNA expression in all tissues at feasible doses, and a bioinformatics effort to provide information in a friendly environment. Thus, an integrative combination of programs executed at different levels (mRNA, lncRNA, circRNA, miRNA) may be envisioned and even a dynamic adaptation according to different time points. Nowadays, considering the low number of known biological functions of mRNAs, let alone non-coding RNAs, the process will be long, and during it, some gene functions will be linked to many dietary components.

The influence of Mediterranean diets or their components on miRNA has only been studied in two reports. No study has addressed the regulation of lncRNA or circRNA expressions. Since olive oil is an important component of this dietary pattern, much effort has been devoted to understanding the effect of MUFA, the major components of this oil. The replacement of SFA with MUFA reduced metabolic stress and prevented the expression of inflammatory genes in different tissues (PMBC, adipocytes, PBMC, coronary artery smooth muscle, and human prostate cancer cells), and consequently to a less atherogenic profile and control of tumor progression when compared to other sources of fat. In animal models, colon and mammary gland tumorigenesis has been found to be really sensitive to this issue. This similar response in different models and experimental conditions suggests a conserved mechanism in different species.

Minor components of virgin olive oil have been particularly studied and different authors have found that the consumption of virgin olive oil with phenolic compounds, either in a postprandial regimen or over a relatively short period of time, influences the expression of genes related to atherosclerosis progression, and that this effect is observed at the moderate doses consumed in this dietary pattern. Whether this action is due to a single or a combination of phenolic compounds awaits an answer. Not much effort has been devoted to the translation of theses transcriptional changes into proteins. However, in vitro studies clearly indicate that extracts enriched in hydroxytyrosol, secoiridoids, and lignans are able to inhibit cell proliferation by modulating cell cycle expression. We need to explore whether these compounds act synergistically, as well as their action in controlled clinical trials.

Despite their being more abundant than phenolic compounds in virgin olive oil, terpenes have received less attention. In animal models, oleanolic and maslinic acids were particularly active in controlling the circadian clock and inflammation genes, respectively. Thus, the term “MUFA-rich oil” no longer appears appropriate for encompassing all the oils to which it is currently applied, paying no attention to the active minor components (phenolic and terpenic compounds).

The results reviewed provide at least a partial molecular basis for the reduced risk of cardiovascular disease and cancer observed in Mediterranean countries. The nutrient complexity of the Mediterranean diets, differences among the studies and the diversity in the responses depending on the specific genetic makeup makes this field an open arena with enormous possibilities. Increasing and consolidating the nutrigenomic knowledge of this diet warrants further research in order to provide sound, personalized, and optimized nutritional recommendations.

## Figures and Tables

**Table 1:** Combinations of keywords used to search the PubMed database.

Olive Oil	Number of References	Mediterranean Diet	Number of References
DNA arrays	1	DNA arrays	1
Microarray	28	Microarray	7
Microarrays	11	Microarrays	3
Gene expression profile	26	Gene expression profile	11
Transcriptional profile	11	Transcriptional profile	6
RNA profile	8	RNA profile	1
Transcriptome	19	Transcriptome	12
Transcriptomics	2	Transcriptomics	12
RNA seq or RNAseq	0	RNA seq or RNAseq	1
mRNA-Seq	1	mRNA-Seq	1
RNA sequencing	1	RNA sequencing	0

Accessed on 7 April 2017.

**Table 2:** Study characteristics.

Species	Tissue	Platform	Number of Probes	Dietary Intervention	References
Human studies					
Ex vivo	Adipose tissue	GeneChip arrays Affymetrix	17,699	MUFA vs. SFA	[20]
Ex vivo	PBMC	PrimeView ™ arrays Affymetrix	36,000	Med diet vs. previous diet	[21]
Ex vivo	PBMC	GeneChip arrays Affymetrix	17,699	MUFA vs. SFA vs. Med diet	[22]
Ex vivo	PBMC	Oligo Microarrays (G4112A) Agilent	45,220	Phenolic compounds, postprandial	[23]
Ex vivo	PBMC	HT-12 v4 Illumina V2 MicroRNA Illumina	47,000	Phenolic compounds, postprandial	[24]
			1146		
Ex vivo	PBMC	Gene 1.1 ST Array Affymetrix	56,249	MUFA vs. SFA, postprandial	[25]
Ex vivo	PBMC	Genome Survey Microarray V2.0 Applied Biosystems	32,878	Virgin olive oil, postprandial	[26]
Ex vivo	PBMC	GeneChip U133A 2.0 Affymetrix	18,400	Low fat vs. Med diet	[27]
Ex vivo	PBMC	Genome Survey Microarray V2.0 Applied Biosystems	32,878	Long-term virgin olive oil consumption	[28]
Ex vivo	PBMC	HT-12 v4 BeadChip Illumina	47,000	OO vs. EPA or DHA	[29]
Cells of human origin					
In vitro	Colon cancer cells	Custom-made Oligo Microarray chip	700	Phenolic compounds	[30]
In vitro	Erythroleukemic cell line K562 and keratinocytes	RNA seq	20 × 10^6^ reads	Hydroxytyrosol	[15,16]
In vitro	JIMT1 breast cancer cells	Oligo Microarrays (G4112F) Agilent	45,220	Phenolic compounds	[31,32]
In vitro	Mesenchymal stem cells	Serial Analysis of Gene Expression	Counts not shown	Oleuropein	[19]
In vitro	Prostatic tumors	Human genome 133A 2.0 Affymetrix	22,000	Fish oil vs. OO or corn oil	[33]
In vitro	SMC	Low density array	4376	TRL from MUFA vs. SFA vs. Med diet	[34]
Animal studies					
*C. elegans*	Whole organism	*C. elegans* Gene-Chip ^®^ Genome Arrays Affymetrix	22,500	Tyrosol (250 µM)	[35]
Flounder	Liver	Chip Array Tokyo University	14,461	OO vs. LO	[36]
Mouse	Adipose tissue	MouseRef-8 v2 Illumina	25,600	Hydroxytyrosol (0.03%)	[37]
Mouse	Cerebral cortex Cerebelum	Agilent Mouse GE 44K v2 and Agilent mice miRNA array	39,430	Phenolic content of OO	[38]
			1247		
Mouse	Intestine	GeneChip MOE430 2 Affymetrix	39,000	OO vs. palm or safflower	[39]
Mouse	Liver	GeneChip MOE430A Affymetrix	22,690	Unsaponifiable fraction of OO	[40]
Mouse	Liver	GeneChip MOE430A Affymetrix	22,690	Oleanolic acid	[41]
Mouse	Liver	GeneChip MOE430A Affymetrix	22,690	Maslinic acid	[42]
Mouse	Liver	RNA seq	23 × 10^6^ reads	OO vs. Western or Long-chain MUFA	[17]
Rat	Colon	Codelink UniSetRat I GE Healthcare	9028	Fish oil vs. OO or corn oil	[43,44]
Rat	Liver	Array Affymetrix	12,500	Fish oil vs. OO	[45]
Rat	Liver	Rat U34 Affymetrix	26,334	MUFA vs. SFA, long-term	[46]
Rat	Liver	Expression Array 230 version 2.0 Affymetrix	31,000	Virgin olive oil, fasting vs. postprandial	[47]
Rat	Liver	RGU34A GeneChip Affymetrix	8800	OO vs. corn oil, long-term regimen	[48]
Rat	Mammary glands	RNA seq	Counts not shown	OO vs butter or safflower	[18]
Rat	Mammary tumors	Expression Array 230 2.0 Affymetrix	31,000	Hydroxytyrosol	[49]
Rat	Mammary tumors	Rat Exon 1.0 ST Array Affymetrix	1 × 10^6^	OO vs. corn and low fat diets	[50]
Swine	Muscle	Porcine genome array Affymetrix	23,937	OO vs. beef tallow, coconut or soybean oils	[51]

DHA: docosahexaenoic acid; EPA: eicosapentaenoic acid; LO: linseed oil; Med: Mediterranean; MUFA: monounsaturated fatty acids; OO: olive oil; PBMC: peripheral blood mononuclear cells; SFA: saturated fatty acids; SMC: smooth muscle cells; TRL: triglyceride-rich lipoproteins.
